# Optimization of Roasted Green Tea Winnowing via Fluid–Solid Interaction Experiments and Simulations

**DOI:** 10.3390/foods11203271

**Published:** 2022-10-20

**Authors:** Kun Luo, Chengmao Cao, Zhengmin Wu, Xuechen Zhang, Minhui An

**Affiliations:** 1 School of Engineering, Anhui Agricultural University, Hefei 230036, China; 2 State Key Laboratory of Tea Plant Biology and Utilization, Anhui Agricultural University, Hefei 230036, China

**Keywords:** tea particle motion, flow field, fluid–solid interaction, tea modeling, winnowing mechanism

## Abstract

In the tea industry, achieving a high winnowing accuracy to produce high-quality tea is a complex challenge. The complex shape of the tea leaves and the uncertainty of the flow field lead to the difficulty in determining the wind selection parameters. The purpose of this paper was to determine the accurate wind selection parameters of tea through simulation and improve the precision of tea wind selection. This study used three-dimensional modeling to establish a high-precision simulation of dry tea sorting. The simulation environment of the tea material, flow field, and wind field wall were defined using a fluid–solid interaction method. The validity of the simulation was verified via experiments. The actual test found that the velocity and trajectory of tea particles in the actual and simulated environments were consistent. The numerical simulations identified wind speed, wind speed distribution, and wind direction as the main factors affecting the winnowing efficacy. The weight-to-area ratio was used to define the characteristics of different types of tea materials. The indices of discrete degree, drift limiting velocity, stratification height, and drag force were employed to evaluate the winnowing results. The separation of tea leaves and stems is best in the range of the wind angle of 5–25 degrees under the same wind speed. Orthogonal and single-factor experiments were conducted to analyze the influence of wind speed, wind speed distribution, and wind direction on wind sorting. The results of these experiments identified the optimal wind-sorting parameters: a wind speed of 12 m s^−1^, wind speed distribution of 45%, and wind direction angle of 10°. The larger the difference between the weight-to-area ratios of the tea leaves and stems, the more optimized the wind sorting. The proposed model provides a theoretical basis for the design of wind-based tea-sorting structures.

## 1. Introduction

Green tea is an important economic crop in China. The role of green tea in human health is becoming increasingly evident, and the demand for high-quality tea is soaring [1]. Roasted green tea is produced during spring, summer, and autumn, resulting in high volumes of product year-round. However, low prices have always constrained the development of green tea production. The process of grading enables the production of high-quality tea leaves of uniform shape and weight, increasing the value of each kilogram of roasted green tea by a factor of 3–5 [2]. Hence, high-precision sorting of tea leaves is essential for improving the value of tea. During the sorting of the tea materials in this study, the color-sorting equipment was inefficient and did not sort materials with similar color. The mesh-sorting equipment was readily blocked by the tea materials, and it simultaneously sorted tea stems and tight leaves. The high price of electrostatic sorting equipment and the uneven shapes of the sorted tea leaves were not suitable for sorting the materials in this study [3]. At present, high-volume tea sorting primarily relies on wind-sorting equipment, which is widely used for its high efficiency, low cost, and adaptability. However, the parameters of the currently available wind-based tea-sorting structures rely on manual empirical design. Furthermore, the parameters of the wind-sorting process cannot be precisely configured for different types of tea. These problems result in unacceptably low quality in the wind-sorting process [4]. For this reason, it is important to explore the theoretical basis of efficient wind-based tea sorting to promote the global production of high-quality tea.

In previous studies, the modeling of tea particles via the equivalent ball model could not accurately represent a real winnowing environment. In addition, simulations of the flow field have been used to separately analyze the particles and flow field, but these simulations were limited in their application. Therefore, further studies on the influence of the particles on the flow field and vice versa, as well as the optimization of these factors, are required [3]. To improve the grading accuracy of fresh machine-picked tea leaves, the key parameters of fresh leaf wind selection were obtained based on the analysis using the numerical calculation method of the two-phase flow between machine-picked fresh leaves and airflow [4]. Zhong et al. [5] analyzed the flow field based on hydrodynamics and found that the installation of spoilers resulted in a more stable flow-field distribution. The effective drift distance is the greatest when the velocity distribution of the wind inlet is small at the top and large at the bottom.

The modeling of small particles that aggregate in the shape of the tea materials can yield results that are more similar to a real winnowing environment via the model-fitting capability of discrete element software. For example, Zhu et al. [6] used EDEM and FLUENT software to study the centrifugal water-washed tea dehydration process and analyze the influence of the wind speed, centrifugal force, and screw propeller on the dehydration. Zhao et al. [7] used numerical calculations and EDEM simulations to analyze the kinematics of the spherical tea-roasting process, which enabled the key parameters of the roasting equipment to be determined. In addition, the computational fluid dynamics (CFD) method was used to simulate the withering process of black tea, and the effects of the position of the gas path, height of the withering machine, and spatial distance on this process were examined. They also used the EDEM simulation method to study the key structures of the fresh tea leaf classifier, and the speed and inclination parameters of the cone drum were determined [8]. Furthermore, the EDEM–FLUENT process has been used to study the influence of temperature distribution on the tea-curing process, analyze the temperature distribution and heat transfer efficiency, and determine a strategy for generating the optimal temperature structure [9]. Most previous studies on tea simulations focused on fresh leaves and utilized a simplified equivalent sphere model. However, the shape and structure of the tea leaves significantly influence the results of the winnowing process, necessitating an analysis of the dynamics of wind-based tea sorting using three-dimensional scanning technology and simulations of the relevant interactions.

However, previous studies have exhibited several limitations. First, the different aerodynamic parameters of tea materials (i.e., stems and leaves) strongly influence the way they drift. However, in previous studies, the physical parameters of tea materials were assumed to be similar. In fact, the aerodynamic parameters of tea materials vary owing to the influence of factors such as their condition and the plantation environment. This adds considerable uncertainty to the quantitative analysis of the relationship between different tea materials and the flow field [10]. Second, previous studies focused on the analysis of the wind field and the requirements for tea material sorting, given the combined effects of the wind direction, wind speed, weight-to-area ratio of the tea materials, and discrete degree. This warrants further exploration in more detail [11]. Third, at present, the adjustment of the winnowing parameters relies on experience. Research on the aerodynamic interaction between tea materials and the flow field provided a theoretical basis for the design of an adaptive control system for wind-based tea sorting [2].

The purpose of this study was to determine the accurate wind speed, wind angle, and separation baffle position parameters for tea wind selection. In this paper, a three-dimensional high-precision modeling method was used to establish the tea model. Modeling of tea wind selection was carried out using a fluid–solid interaction simulation method. Simulation tests and test benches were used to determine the factors affecting tea wind selection. Finally, the exact parameters of tea wind selection were verified with the help of practical tests. This study was used to improve the efficiency and accuracy of tea wind selection.

## 2. Materials and Methods

### 2.1. Physical Properties of Materials

Green tea from the Changqing Tea Factory in Xuancheng, Anhui Province, China, was selected as the dry tea material for this study. This tea mainly comprised stems, large leaves, and compact leaves. Tea samples (50 g) were randomly selected, and then they were dumped into a 1.5 mm sieve to remove dust, broken residues, and other impurities.

#### 2.1.1. Determination of the Weight-to-Area Ratio

The weight-to-area ratio was defined as the ratio of the weight of a tea material to its windward area. The larger the area, the stronger the force of the wind on the material. Tea leaves have similar areas and, therefore, experience approximately the same wind force. However, differences in weight cause variations in the drift distance. The formula for the weight-to-area ratio is [12]

(1) $$R_{A}=\frac{M}{A}$$

where *M* is the weight of the tea (in kg), and *A* is the tea windward area (in m^2^). The accurate measurement of the weight-to-area ratios of the different tea materials was important for analyzing their trajectories. The area represents the interface over which the tea material comes into contact with the wind, and the actual measurement corresponded to the largest projected area of the tea leaves. The weight was measured using an electronic balance (YP203B, Shanghai Puchun Metrology Instrument Co., Shanghai, China) with an accuracy of 0.001 g.

#### 2.1.2. Density and Relative Composition

**Figure 1:**
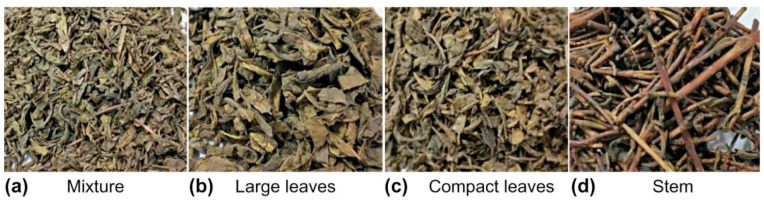
Composition of dried green tea.

The density of the tea materials affects their trajectories in the flow field. Thus, the densities of each type of material in the tea were obtained using a specialized instrument (WKT-300A, Jiangsu Ketai Instrument Co., KunShan, China). The quality and relative composition of the tea stems, large leaves, and compact leaves for each 50 g sample were determined. The material samples are shown in Figure 1, and the statistical parameters are presented in Table 1.

### 2.2. Working Principles

#### 2.2.1. Adaptive Wind-Sorting Structure

**Figure 2:**
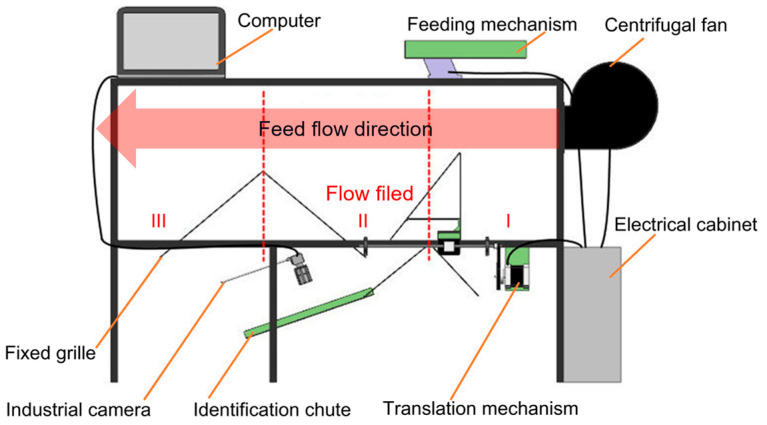
Structure of adaptive winnowing machine. Locations labeled “I”, “II”, and “III” represent adjustable stem collection area, adjustable leaf collection area, and broken leaf collection area, respectively.

Figure 2 shows the adaptive wind-sorting test bench, which consisted of a variable speed fan, adjustable grid, wind chamber, identification system, and feeding mechanism. The material was evenly inserted into the wind chamber through the feeding mechanism, and the force of the wind caused the tea material to drift along the length of the wind chamber. The material falling into area II entered the identification chute via grid collection, and the identification system determined the relative proportion of stems and leaves in the collection area. The output parameters of the control function model determined the grid position and wind speed. After multiple cycles, the relative proportion of stems and leaves tended to be optimal. To maximize the effectiveness of the adaptive wind-sorting system, the aerodynamic characteristics of the different tea materials were analyzed. The results of this analysis allowed the functional relationship between the wind speed, wind direction, grid position, and optimal stem-to-leaf ratio to be determined.

In addition, determining the relationship between the wind speed, weight-to-area ratio, and drift trajectory was essential for adaptive wind sorting. For this purpose, the grid was placed at the outlet of the fan, and the density of the grid was adjusted to enable the wind speed of each layer in the outlet to be distinguishable. The rotating baffle at the outlet adjusted the wind direction. The high-speed air flowed over the tea materials, which began to separate according to the combination of wind and gravity. The force of the wind on each type of material determined the drift distance. The preliminary analysis of the flow field showed that the wind speed was distributed in a stepwise manner and that the effect of the wind sorting was optimal when the wind direction angle was 16°. However, the optimal wind speed varies because of the different weight-to-area ratios of each tea sample. Determining the relationship between wind speed, weight-to-area ratio, and drift trajectory is essential to achieving adaptive wind selection.

#### 2.2.2. Mathematical Modeling

In this study, the CFD-discrete element method (DEM) model was used to describe the motion and distribution process of tea leaves in the flow field. The movement and collision of tea leaves during the distribution are tracked by time stepping simulation using DEM. The motion of the tea particles is controlled by Newton’s second law of motion, and the equation of motion [13] is

(2) $$m_{i}\frac{dv_{e,i}}{dt}=F_{pf,i}+\sum_{j=1}^{k_{i}}\left(F_{n,ij}+F_{t,ij}\right)+F_{w,j}+m_{i}g\;$$

(3) $$I_{i}\frac{d\omega_{e,i}}{dt}=\sum_{j=1}^{k_{i}}\left(T_{t,ij}+T_{r,ij}\right)+T_{w,i}$$

where the subscript *i* represents particle properties; $m_{i}$ is mass, kg; $m_{i}g$ is the gravity, N; $v_{e,i}$ is the translational velocity, m s^−1^; $F_{pf,i}$ is the particle–fluid interaction force, N; $F_{w,i}$ is the contact force between the wall and particle, N; $F_{n,ij}$ is the normal force between the particles, N; $F_{t,ij}$ is the tangential force between the particles, N; $I_{i}$ is the moment of the inertia of the particle, kg m^−2^; $\omega_{e,i}$ is the rotation velocity, rad s^−1^; $T_{t,ij}$ is the tangential torque between the particles; and $T_{r,ij}$ is the rolling friction torque between the particles, N s^−1^.

The gas phase is regarded as a continuous phase, which conforms to the laws of conservation of mass and momentum. In this paper, the control equation of the standard k-epsilon model was chosen. The control equation [14,15,16,17] is expressed as

(4) $$\frac{\partial \left(\varepsilon \rho \right)}{\partial t}+\nabla •\left(\varepsilon \rho v\right)=0$$

(5) $$\frac{\partial \left(\varepsilon \rho v\right)}{\partial t}+\nabla •\left(\varepsilon \rho v\right)=-\varepsilon \nabla p+\nabla •\left(\varepsilon \tau \right)+\varepsilon \rho g-F_{p}$$

where ρ is the air density (kg/m^3^); ε is the void fraction; v is the velocity (m/s); τ is the fluid viscous stress tensor (Pa); and $F_{p}$ is the interaction force (N).

### 2.3. Fluid–Solid Interaction Simulation

#### 2.3.1. Particle and Flow Modeling

**Figure 3:**
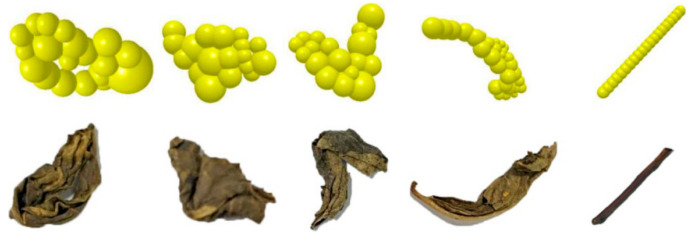
Simulation models for various shapes of tea materials.

Based on previous interaction simulations in the agricultural field, this study designed an interaction simulation of wind-based tea sorting according to the characteristics of tea [18,19,20]. The electronic file containing the three-dimensional grid for tea was imported into EDEM, and the tea model was stacked using multiple equivalent balls. In addition, a 3D scanner (Handyscan 700, Creaform, Leavis, Quebec, Canada) was used to establish a three-dimensional model of the tea materials. After the scanning was finished, the point cloud model was obtained, and the model was repaired using the following software. UG software was used to convert the format of the repaired model. Next, ANSYS 17.0 was used to divide the grid and generate the grid file. Subsequently, a three-dimensional model of the tea materials was stacked using EDEM [18]. The minimum and maximum particle radii were 0.5 mm and 1.5 mm, respectively. In the interaction field, the three-dimensional trajectory of the material was analyzed. Five tea material samples were selected according to their weight-to-area ratios, and their models are shown in Figure 3. The models were simplified to obtain a balance between the accuracy of the calculation and the time required to execute it. The requirements of the simulation in terms of area, weight, and roughness were met.

**Figure 4:**
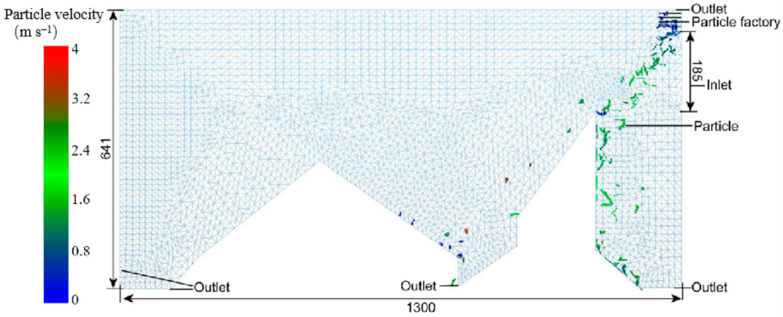
Simulation flow field (units: mm).

The interaction flow field was used to analyze the influence of wind speed, wind direction, and fluid distribution on particle motion. The simplified flow field model is shown in Figure 4. There were differences in the shapes, densities, and weight-to-area ratios of the different tea materials. To effectively distinguish between the leaves and stems in the flow field, it was essential to accurately match the wind speed to the wind direction. Therefore, the flow-field simulation model considered only the contact area between the material and flow field and disregarded the material, wind source, and collection area.

The EDEM model consisted of a particle factory and geometry. There were three outlets in the lower part of the EDEM model, and the upper and right sides were airflow outlets. The width of the model was 106 mm, the length of the wind inlet was 185 mm, the length of the geometry model was 1300 mm, and the height was 641 mm. The particle plant was set in the upper right corner with a width of 50 mm and length of 100 mm. The height of the right-hand grille and the distance from the right-hand wall were determined from subsequent experimental analysis. The FLUENT model featured a flow-field area, wall, fluid inlet, and fluid outlet. EDEM and FLUENT shared a grid model, and they were both used to simulate the motion state of the particles. A user-defined function (UDF) was used to transmit the simulation data from each step of the two software packages.

#### 2.3.2. Optimal Design of Wind Field

The wind speed and direction used in existing wind-based tea-sorting machinery are fixed, and the degree to which leaves and stems are separated is decreased when teas with different weight-to-area ratios are sorted [21]. To improve the adaptability and accuracy of the wind-based tea-sorting machinery used in this study, specific wind field parameters needed to be assigned according to the characteristics of the tea material. The primary parameters of the wind-sorting structure were the wind direction, wind speed, wind speed distribution, and grid position. The first three were the main factors that determined the distribution of the tea materials. By changing the values of these three parameters, the degree of separation between different types of stems and leaves reached a reasonable value.

**Figure 5:**
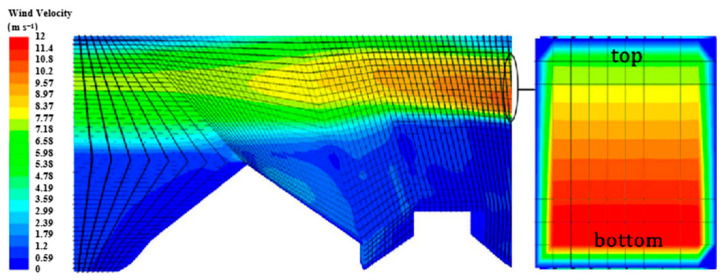
Structure of wind field.

In the simulation, only the flow-field inlet, flow-field outlet, and flow-field wall were retained in the wind field, and the grid was removed to analyze the drift trajectory of the tea material. The test materials were obtained after filtering out the dust and foam. The influence of wind speed, wind direction, and wind speed distribution on the drift of the material was analyzed. The wind field structure, which is shown in Figure 5, was designed on the basis of wind-sorting structures from previous studies. To explore the influence of the flow field on the separation, drift trajectory, and distribution position of the tea material, the wind speed inlet function was determined using a UDF expressed as

(6) $$V_{y}=b-ky$$

where the slope *k* reflects the distribution of the wind speed at different heights *y* of the wind speed inlet, and the constant *b* represents the maximum wind speed. The overall wind direction was changed by setting the wind speed inlet vector. The lowest and highest wind speed values at the wind speed inlet were substituted into the formula to obtain the value of *k*.

The wind speed distribution is a percentage of the top and bottom wind speed values, as shown in the wind speed inlet on the right in Figure 5. The wind speed distribution equation is expressed as follows:

(7) $$V_{d}=\frac{v_{top}}{v_{bottom}}•100\%$$

where *V_d_* represents the wind speed distribution, *v_top_* represents the top wind speed, and *v_bottom_* represents the bottom wind speed.

#### 2.3.3. Interaction Parameter Settings

The parameters of the particles and geometry were set in EDEM. In this experiment, particle–particle and particle–geometry collisions were disregarded. The material of the geometry wall was defined as steel. The contact coefficient and recovery coefficient of the particles and geometry are presented in Table 2 [22]. The particle factory was placed inside the geometry and randomly generated tea particles according to statistical distributions. The time step in FLUENT was set to 10 times that set in EDEM [14]. The minimum volume of the flow-field mesh was 5.4 × 10^−7^ m^3^, which was significantly larger than the particle size. The particle motion was controlled by the flow-field simulation. After one simulation was performed, the simulation information in FLUENT and EDEM was swapped [23].

Next, the flow-field parameters were set in FLUENT, and the turbulence model was used. The inlet, outlet, and wall of the flow field were assigned according to the actual test bench. The inlet of the flow field was a velocity inlet, and the outlet was a pressure outlet. Wind speed conditions were determined based on actual experiments. The UDF was customized to realize the direction, magnitude, and distribution of wind speed conditions. The simulation primarily considered the effect of the flow field on the particles. Therefore, in this study, the Poisson’s ratio for the tea stems was consistent with that of the tea leaves [12].

This model solved the fluid motion process. In particular, the model uses a velocity inlet and pressure outlet. In this study, only the velocity, trajectory, and forces on the tea particles were analyzed. Therefore, in EDEM, we selected the Hertz–Mindlin model. This study examined the state of fluid motion at each point in space.

### 2.4. Simulation and Experimental Error Analysis

#### 2.4.1. Comparison between Tea Material Trajectories in Simulation and Experiments

**Figure 6:**
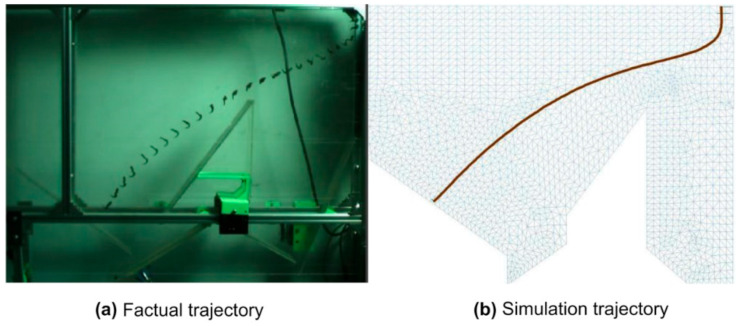
Test trajectory of tea material. (**a**) Actual trajectory captured using a high-speed camera. (**b**) Trajectory tracked by postprocessing software.

The trajectories of the tea materials in the actual and simulated wind fields are shown in Figure 6. In the experiment, the wind speed sensor (AR866A, WanChuang Electronic Products Co, Dongguan, China) was used to measure the wind speed at each point in the wind speed inlet, and the wind speed was linearly distributed from the top to bottom of the inlet (the function of which was obtained through statistical analysis). The inlet boundary conditions of the wind speed were compiled using the UDF interface of FLUENT software, and the measured wind speed function was used as the simulation parameter. The motion of the tea material in the wind field was recorded using a high-speed camera (i-SPEED3, OLYMPUS, Tokyo, Japan), as shown in Figure 6a, and postprocessing software was used to track the trajectory, as shown in Figure 6b. The maximum frame rate was 10,000 fps. Moreover, in this experiment, 200 fps was used. The results showed that the tea material moved along a parabola after free fall, and the trajectory was in good agreement with the distance data. The deviation between the experiment and simulation was reflected by the difference between the vertical coordinates at the same horizontal position. The validation error was the average deviation of the two longitudinal coordinate values. The error for the tea trajectory was 10 mm. Moreover, the influence of the error value on this experiment was negligible.

#### 2.4.2. Speed Change of Tea Material

**Figure 7:**
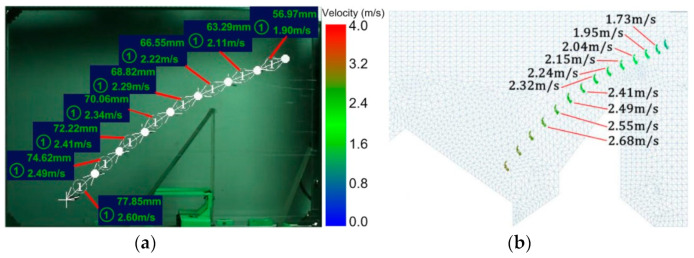
Velocity diagram derived from (**a**) experimental test and (**b**) simulation.

Figure 7 shows the time-dependent velocity of the tea material in the experiment and simulation. The high-speed camera obtained the actual tea material motion, and the software analyzed the velocity values of each segment. The actual speed of the tea material gradually increased, as shown in Figure 7a, and the velocity value of each segment was obtained using EDEM postprocessing software, as shown in Figure 7b. The simulated velocity calculations were similar to the velocities measured by the image processing software, and the trends in the velocity changes were consistent. In summary, the parameters determined by analyzing the tea material motion and changes in the flow field were reliable. Therefore, the simulation model could be used for the subsequent simulation of tea materials to study the influence of the wind direction, wind speed, and weight-to-area ratio on the drift distribution of the tea materials. The deviation between the experiment and simulation was reflected by the difference between the velocities at the same horizontal coordinate position. The validation error was the average deviation of the two velocity values. The error value for the instantaneous velocity was 0.14 m s^−1^. Moreover, the influence of the error value on this experiment was negligible.

#### 2.4.3. Comparison of Simulated and Experimental Wind Speed Inlet

**Figure 8:**
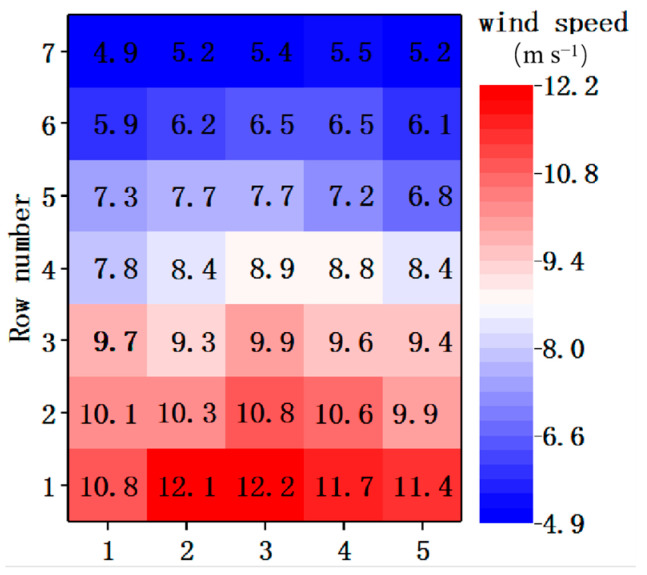
Grid of wind inlet velocities.

The consistency of the wind speed between the simulated and experimental wind speed inlets determined the accuracy of the final results. In this study, screens were used to adjust the airflow from the fan. Different layers and shapes of screens allowed the air velocity to reach the nominally designed value at each point. The air outlet was divided into a uniform grid with 35 sections, as shown in Figure 8. Using wind speed sensors for each section, the speed of the fans was measured at a constant voltage. The number of layers of mesh and the density of the meshes are constantly adjusted to achieve a uniform wind speed. The relative standard deviations for each row of data were all less than 6%, and the decreasing trend in the data for each column was the same as the trend defined in the simulation. The deviation between the experiment and simulation was reflected by the difference between the wind speeds at the same height. The validation error was the average deviation of the two wind speed values. The error value for the wind speed inlet was 0.37 m s^−1^. Moreover, the influence of the error value on this experiment was negligible. Therefore, the experimental wind speed inlet met the requirements of the design.

## 3. Results and Discussion

### 3.1. Evaluation Indicators

#### 3.1.1. Drift Limit Velocity

The velocity of tea materials in the flow field continuously increased, and the velocity of the tea materials in contact with the grid represented the limit of the drift velocity. Excessive drift velocities cause damage to tea materials [12]. Moreover, a greater drift speed increases the drift distance [24]. The linear velocity of the tea material was consistent with the trend in the kinetic energy. The interaction simulation was used to monitor the speed of the tea materials in real time, which enabled the critical drift velocity of the tea materials to be obtained.

**Figure 9:**
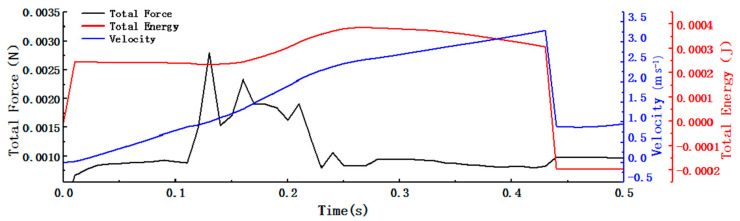
Force (black line), kinetic energy (red line), and velocity (blue line) of tea material.

Figure 9 shows the changes in the velocity, force, and kinetic energy that occurred while the tea materials drifted. As shown in the figure, the speed and kinetic energy of the tea materials first increased and then rapidly decreased after the tea materials struck the baffle. Compared with the trajectory in Figure 6, the speed of the tea materials slowly increased under the influence of gravity and the wind. The speed of the tea materials rapidly increased when the tea leaves fell through the area containing a high wind speed, and it slowly increased when they fell out of that area. The velocity rapidly decreased after the tea materials impacted the baffle. The trends in the tea drift velocity and total kinetic energy were the same as those in the trajectories of the tea materials. With a decrease in the tea drift limit velocity, the tea was subject to less damage. In addition, the drift distance decreased. When the tea drift limit velocity and drift distance decreased, the sorting effect was improved.

#### 3.1.2. Drag Force Difference

**Figure 10:**
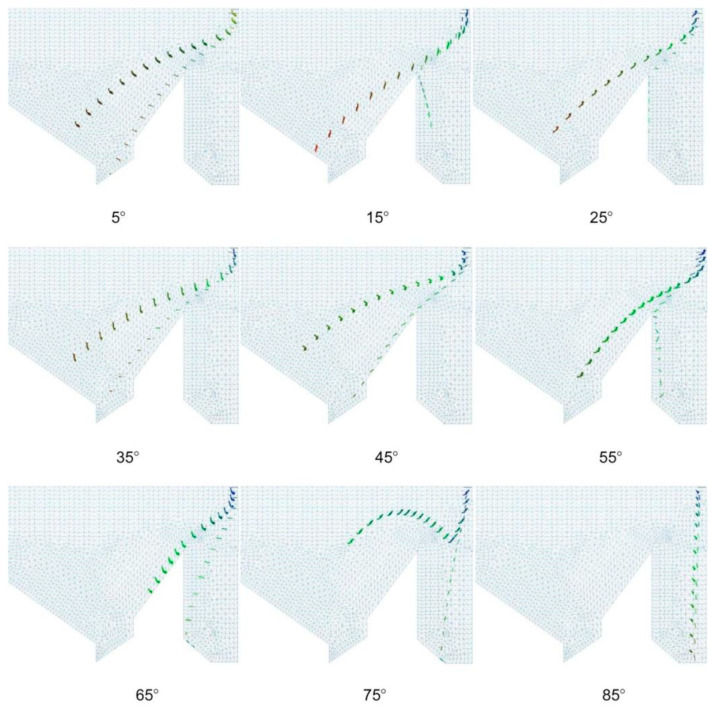
Leaf (upper trajectory) and stem (lower trajectory) drifts resulting from various wind direction angles.

The wind caused the tea to drift forward, and the drag force dispersed the agglomerated tea material. The drag force slowed the downward kinetic energy of the tea materials; the greater the drag force at the same height, the farther the tea drifted. The effect of drag on the leaves and stems following the change in the wind direction angle is shown in Figure 10. The upper trajectory represents the leaves, and the lower trajectory represents the stems. The figure demonstrates that, as the wind direction angle increased, the drift distance of the tea material increased. Furthermore, as the angle increased, the separation of the stems and leaves initially improved and then worsened. When the angle was too large, the drift distance of the leaves decreased, and a large number of leaves were lost. Therefore, an angle of 5–25° was selected. As the drag force increased, the drift distance between the tea leaves and stems increased. The greater the difference in the drag force between the tea leaves and stems, the greater the separation.

#### 3.1.3. Discrete Degree

**Figure 11:**
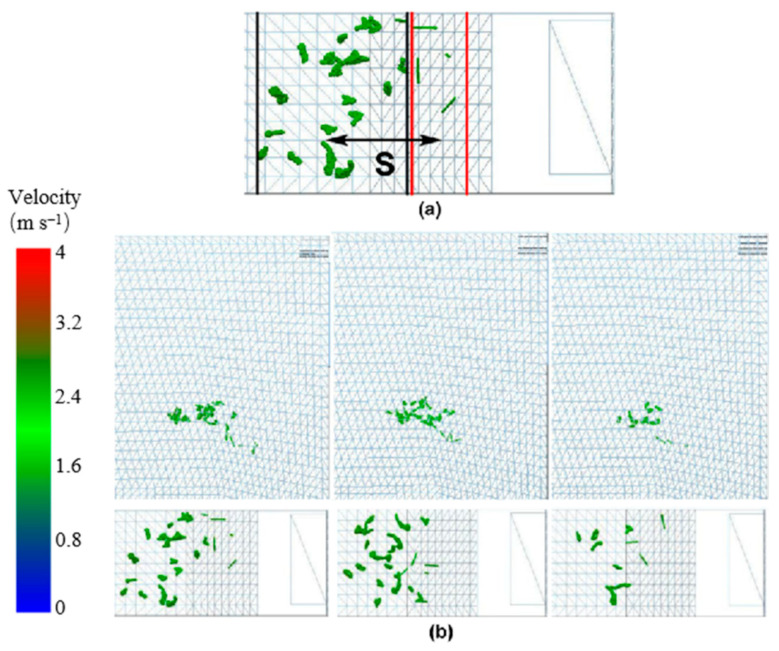
Discrete tea material distribution. (**a**) *S* is the distance from center of leaf distribution to center of stem distribution. (**b**) Distribution of particles in the flow field after the grid plate was removed. In (**b**), top three drawings represent the main views, and the bottom three drawings represent the corresponding top views.

The separation distance between the stems and leaves was regarded as the discrete degree when the tea materials left the acceleration zone. As shown in Figure 11a, the distance S from the center of the leaf distribution to the center of the stem distribution formed a discrete degree.

Figure 11b shows the distribution of the particles in the flow field after the grid plate was removed. The upper part of the figure represents the main view, and the lower part represents the vertical view. The boundary between the stems and leaves was observed in the vertical view. The tea materials fell from the inlet, and it accelerated to a drifting speed owing to the wind. The statistics of the leaves and stems in the same height distribution showed that the smaller the overlap between the stem and leaf distributions, the higher the discrete degree. Following a decrease in the amount that was fed into the sorter, the overlapping interval between the stems and leaves decreased. After the airflow accelerated, the stems with large weight-to-area ratios drifted a short distance, and the leaves with small weight-to-area ratios drifted a long distance. Owing to the influence of the angle of the material as it entered the wind field, three statistical averages were calculated as the final results in each group of experiments.

### 3.2. Analysis of Simulation Results and Discussion

Orthogonal experiments were conducted to analyze the primary and secondary factors affecting the separation, trajectory, and distribution of the tea materials and to determine the optimal combination of parameters. The wind speed distribution, wind speed, and wind direction were examined as experimental variables, and the discrete degree, drag force difference, and drift limit velocity were selected as the evaluation indices. A three-factor, three-level orthogonal table was used to guide the experiments, as shown in Table 3. Three values for the wind speed distribution (25%, 50%, and 75%) were selected to analyze its influence on the discrete degree of the tea materials. Similarly, three values of wind speed (10 m s^−1^, 12 m s^−1^, and 14 m s^−1^) were selected to analyze its influence on the drift trajectory and distribution position. Finally, three angles for the wind direction (10°, 15°, and 20°) were selected to analyze the influence of the wind force on the decomposition of the tea mass.

#### 3.2.1. Orthogonal Experiments

The test results are shown in Table 4.

The wind speed distribution and wind speed had a significant influence on the drift, and a wind direction angle of 10° was more advantageous in the wind-sorting process. Therefore, a wind speed of 10 m s^−1^ and a wind direction angle of 10° were selected for the single-factor experimental analysis of the wind speed distribution. The wind speed distribution ranged from 25% to 75%, with a gradient of 10%. After determining the optimal wind speed distribution, the wind speed was analyzed using a single-factor experiment. The wind speed ranged from 8.5 to 12 m s^−1^, and the gradient was 0.5 m s^−1^. Finally, based on the optimal wind speed distribution and wind speed, the wind direction was analyzed via a single-factor experiment. The wind direction angle ranged from 5° to 15°, and the gradient was 2.5°, as shown in Table 5.

#### 3.2.2. Wind Speed Distribution Experiment

**Figure 12:**
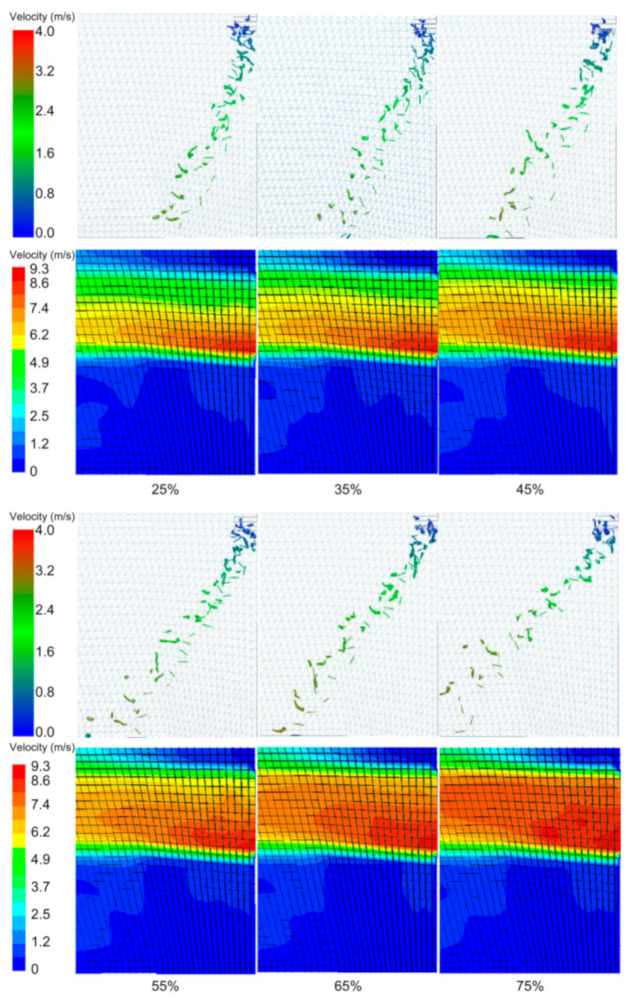
Tea material distributions and corresponding flow-field distributions.

Using a wind speed of 10 m s^−1^ and a wind direction angle of 10°, the influence of the wind speed distribution on the wind-sorting process was analyzed through a single-factor experiment. The tea material drift and flow field for different wind speed distributions are shown in Figure 12. When the wind speed distribution increased, the proportion of high wind speeds at the wind speed inlet gradually increased, and the grouped tea material drifted and gradually separated after leaving the acceleration zone. The changes in the flow field showed that when the wind speed in the upper half of the velocity inlet was too low, the disturbance in the drag force on the grouped tea material was undetectable. Moreover, when the proportion of high wind speeds was too small, the drag force on the grouped tea material was too small to cause stratification. Following an increase in the proportion of high wind speeds, the separation effect became clearer.

**Figure 13:**
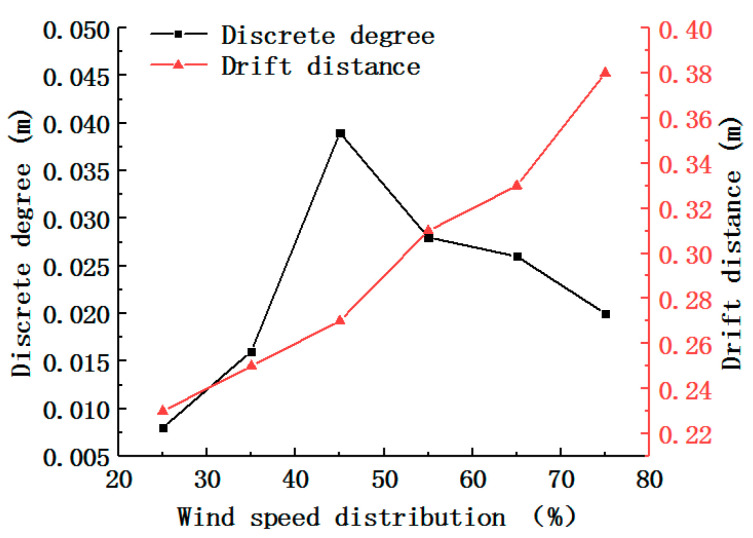
Experimental results for different wind speed distributions.

The results for the discrete degree and drift distance are shown in Figure 13. Following the increase in the wind speed distribution, the discrete degree initially increased and then decreased, reaching its maximum value at a wind speed distribution of 45%. The drift distance increased after the wind speed distribution increased, which was attributed to the continuous action of the drag force on the grouped tea material. An increase in the drift distance led to an increase in the length of the wind-sorting field, but the high length of the wind-sorting field increased the interval over which the wind sorting was invalid. This test mainly considered the separation degree requirement and then considered the processing cost. The wind speed distribution is 45%. This finding is similar to a previous study [5]. However, the predecessors only considered the distribution of the flow field and did not analyze the coupling between the tea particles and flow field. The flow field is disturbed by the disturbance of tea leaves. Therefore, future work can focus on how to control the wind field stability.

#### 3.2.3. Wind Speed Experiment

**Figure 14:**
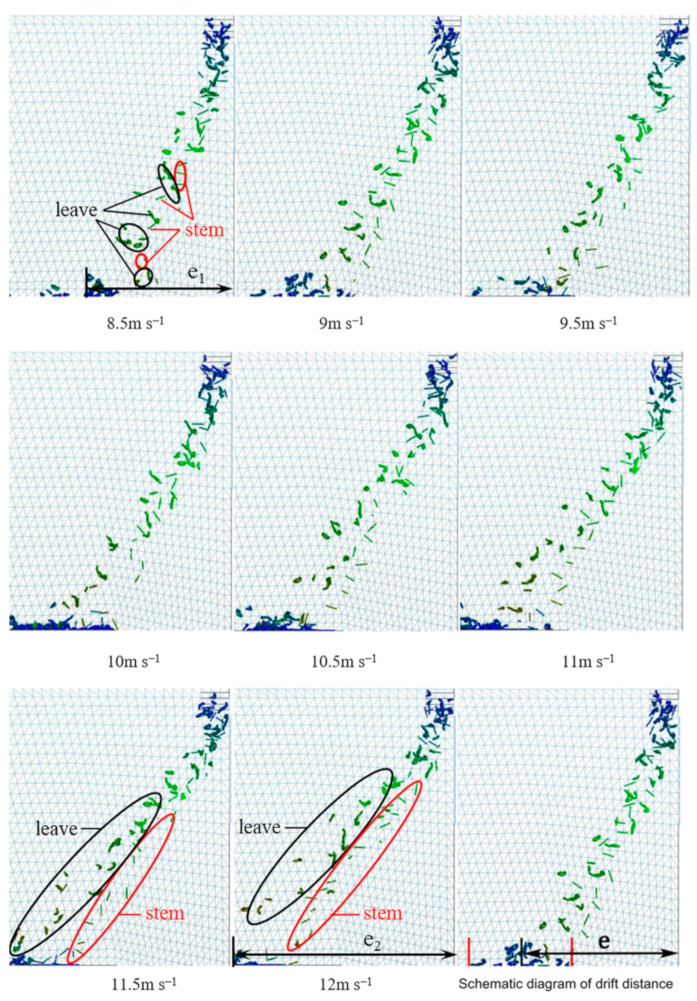
Particle dispersion at different wind speeds.

For a wind speed distribution of 45% and a wind direction angle of 10°, the influence of the wind speed on the wind sorting was analyzed using a single-factor experiment. An adequate separation was evidenced by the clear stratification of the tea leaves and stems. As shown in the lower part of Figure 14, as the wind speed increased, the tea leaves and stalks were clearly stratified. At a wind speed of 8.5 m s^−1^, the tea leaves and stems were not stratified. At a wind speed of 12 m s^−1^, the upper layer contained tea leaves, and the lower layer contained tea stems. Moreover, the interval between their distribution zones increased with an increase in wind speed.

As the wind speed increased, the drift distance of the leaves exceeded that of the stems, and the distribution of the stems became more stable and concentrated than that of the leaves. The ranges over which the materials were gradually distributed were clearly observed. As shown in Figure 14, the drift distance was e_1_ for a wind speed of 8.5 m s^−1^ and e_2_ for a wind speed of 12 m s^−1^. It should be noted that e_2_ is significantly greater than e_1_, thus indicating that the drift distance increased as the wind speed increased. The increase in the wind speed also increased the upper drag force and drift limit velocity. This finding is similar to a previous study [3]. However, the predecessors used round balls with the same particle size instead of tea leaves, which could not respond to the different drift velocities of leaves and stems. The weight-to-area ratio of different materials of tea leaves is similar leading to poor sorting accuracy. Therefore, the difference in the weight-to-area ratio of different materials of tea can be explored to increase in future research. The color of the particles in the image represents the instantaneous speed. The gradual change in color from light green to dark green indicates a gradual increase in speed.

**Figure 15:**
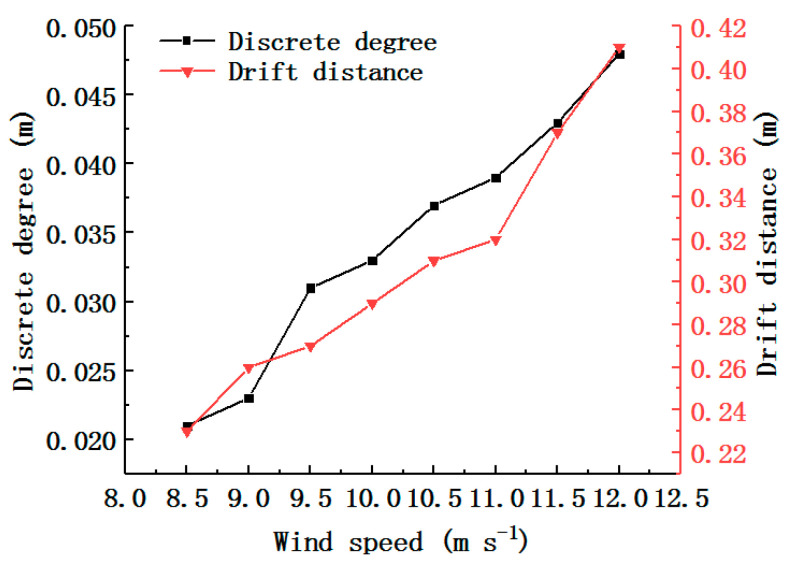
Experimental results for different wind speeds.

Figure 15 shows the results for the discrete degree and drift distance. Both the discrete degree and drift distance gradually increased as the wind speed increased, and they attained their maximum values at a wind speed of 12 m s^−1^. At this wind speed, the drift distance was 0.41 m. When the drift distance exceeded 0.3 m, the invalid area of the wind field led to an excessive wind-sorting structure. To reduce this wind field structure, the height and position of the grid can be adjusted to separate the stem and leaves in advance.

#### 3.2.4. Wind Direction Experiment

**Figure 16:**
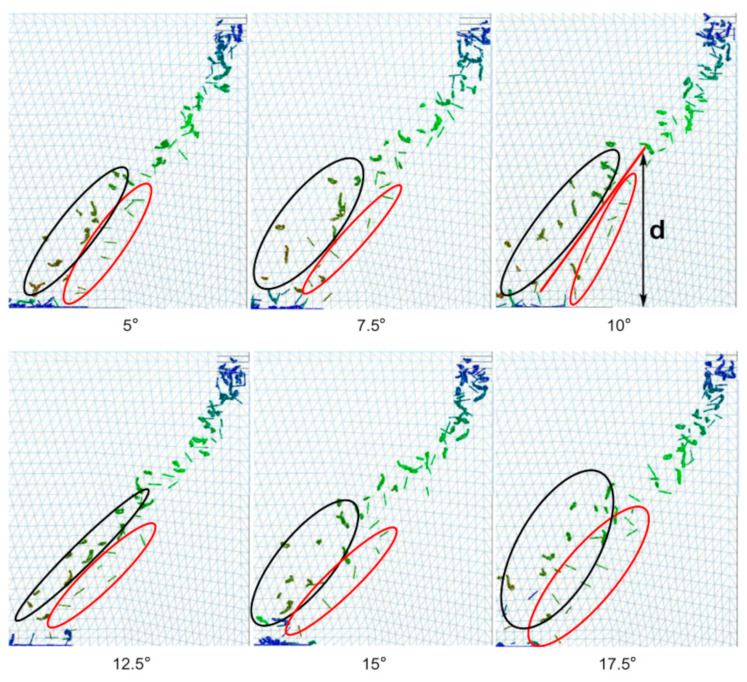
Tea material distributions for different wind directions, where d is stratification height. Black oval is tea distribution area, and red oval is distribution area of tea stems.

After selecting the optimal wind speed distribution of 45% and wind speed of 12 m s^−1^, the influence of the wind direction angle on the wind-sorting process was analyzed using a single-factor experiment. The smaller the overlap between the area of tea leaves in the black oval and the area of tea stalks in the red oval, the better the winnowing effect. The results are shown in Figure 16. As the wind direction angle increased, the dispersion of the grouped tea material initially improved and then worsened. The stratification of the stems and leaves also initially improved and then worsened. A wind direction angle that was too high led to weak stratification. When the wind direction angle was 10°, the dividing line corresponded to the actual stratification, and the distance *d* from the top of the dividing line to the bottom of the image was defined as the stratification height. As the wind direction angle increased, the drift distance did not significantly change. The drift trajectory was affected by the wind direction, and it gradually evolved from the oblique line to the left and then rapidly dropped.

**Figure 17:**
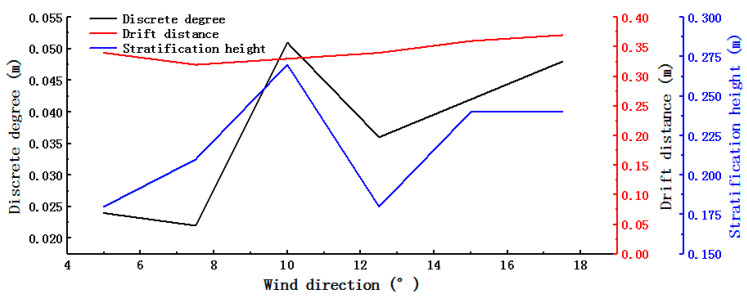
Experimental results for different wind direction angles.

Figure 17 shows the discrete degree, stratification height, and drift distance as a function of the wind direction angle. The trends in the stratification height and discrete degree were very similar. The stratification height reached its maximum value of 0.27 m at a wind direction angle of 10°, at which point the discrete degree was also at its maximum of 0.051 m. Changes in the wind direction angle had a minimal effect on the drift distance. The present finding is similar to the results of a previous study, which showed that wind selection was more effective when the wind angle was 16.3 degrees [24]. However, the tea used by the previous authors was different from this paper, resulting in some differences in the selection of wind selection parameters. Therefore, the accuracy of tea particle modeling determines the accuracy of wind selection parameters. Model accuracy can be improved by reducing the diameter of the model filler particles.

#### 3.2.5. Experimental Results and Discussion

**Figure 18:**
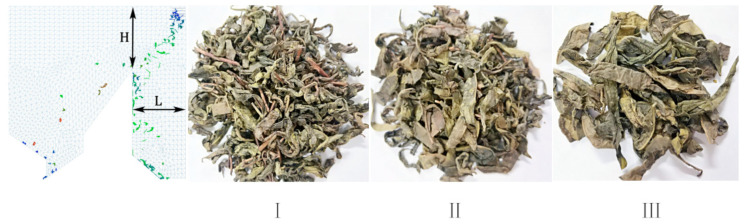
Results of simulation (leftmost panel) and experiments (three right panels). I, II, and III represent collection area.

According to the results of the simulation, the optimal parameters were a wind speed distribution of 45%, wind speed of 12 m s^−1^, and wind direction angle of 10°. These simulation parameters were verified using a wind-based tea-sorting test. In the experiment, 50 g of tea material was selected and inserted into the wind field at a constant speed. The results of the experiment are shown in Figure 18. The grid was set (L) at 0.196 m, and the height (H) was 0.236 m; I, II, and III are the discharge ports.

For different types of tea materials, the greater the difference between the weight-to-area ratio, the better the performance of the wind sorting. The prescreening process, which removes impurities that may affect the wind sorting, can quantitatively change the weight-to-area ratios of the tea stalks and leaves because of their different rates of water absorption and loss. For this reason, wind sorting can be used to effectively separate green tea material containing a large number of leaves and a large number of stalks [4]. Because high-precision discrete element models of different tea materials can enable high-precision wind-based tea-sorting experiments, the simulation model developed in this study can also be used to identify the wind-sorting parameters suitable for other types of tea.

## 4. Conclusions

In order to determine the exact parameters of tea wind selection, a tea flow-solid coupling analysis model was developed in this paper. Simulation models were combined with high-precision tea models to obtain accurate wind selection solutions. Wind-sorting tests were also conducted to verify the performance of the method. The following specific conclusions can be drawn from this study:

(a) The weight-to-area ratio (W/A) has a significant effect on airflow distribution and tea movement. An increase in the difference between the weight-to-area ratio of tea stems and leaves can make the stratification of leaves and stems more obvious.
(b) The wind speed distribution (45%) design of the wind speed inlet can improve the pulling effect of the tea materials. The design can reduce the falling distance of tea leaves. Tea leaves can be quickly dispersed when entering the flow field, which promotes the improvement of wind-sorting accuracy.
(c) When the wind speed is 12 m/s, wind speed distribution is 45%, and wind direction is 10°, the drift distance, stratification height, and dispersion of tea materials all reach desirable levels. More tea stems fell in one zone, and the tea and stems distinction reached the maximum.

## Figures and Tables

**Table 1:** Physical parameters of tea materials.

Material Type	Density (kg m^−3^)	Relative Composition (%)	Weight-to-Area Ratio (kg m^−2^)	Length (mm)	Width (mm)
Stem	455	5.2	0.56–0.79	15–40	1–3
Large leaves	232	36.4	0.35–0.66	28–33	6–10
Compact leaves	250	22.2	0.64–1	15–23	3–5.5
Tattered leaves		34.1			
Dust, foam		2.1			

**Table 2:** Simulation parameters.

	Parameters	Steel	Tea Leaves	Tea Stems
EDEM	Poisson’s ratio	0.25	0.4	0.4
	Density (kg m^−3^)	2500	232/250	455
	Shear modulus (Pa)	1 × 10^8^	2.7 × 10^6^	
	Restitution coefficient (with particles)	0.5	0.36	0.36
	Static friction coefficient (with particles)	0.8	0.1	0.1
	Rolling friction coefficient (with particles)	0.6	0.7	0.7
	Time step (s)	0.000001		
FLUENT	Fluid	Air		
	Inlet velocity function	V_y_ = b−ky		
	Time step (s)	0.00001		

**Table 3:** Values used in orthogonal experiments.

Levels	Wind Speed Distribution	Airflow Direction (°)	Airflow Velocity (m s^−1^)
1	25%	10	10
2	50%	15	12
3	75%	20	14

**Table 4:** Results of orthogonal experiments.

No.	Wind Speed Distribution	Airflow Direction (°)	Airflow Velocity (m s^−1^)	Drift Limiting Velocity (m s^−1^)	Drag Force Difference (N)	Discrete Degree (m)
1	1	1	1	2.9	0.00165	−0.01
2	1	2	2	2.8	0.00075	0.0059
3	1	3	3	2.8	0.002	0.034
4	2	1	2	3.1	0.00189	0.028
5	2	2	3	3.3	0.00189	0.061
6	2	3	1	2.8	0.00121	−0.005
7	3	1	3	4.1	0.00125	0.02
8	3	2	1	3.2	0.00176	0.042
9	3	3	2	3.3	0.002	0.067
Drift limiting velocity	K_1_	2.833	3.367	2.967		
	K_2_	3.067	3.1	3.067		
	K_3_	3.533	2.967	3.4		
	R	0.7	0.4	0.433		
	Primary and secondary factors B > C > A; optimal parameter combination B_3_C_1_A_1_					
Drag force difference	K_1_	0.001	0.002	0.002		
	K_2_	0.002	0.001	0.002		
	K_3_	0.002	0.002	0.002		
	R	0.001	0.001	0		
	Primary and secondary factors A = B > C; optimal parameter combination A_2_B_1_C_1_					
Discrete degree	K_1_	0.01	0.015	0.009		
	K_2_	0.028	0.036	0.034		
	K_3_	0.045	0.032	0.041		
	R	0.035	0.021	0.032		
	Primary and secondary factors A > C > B; optimal parameter combination A_3_C_3_B_2_					

**Table 5:** Parameters used in single-factor experiments.

Parameter	Values							
Wind speed distribution	25%	35%	45%	55%	65%	75%		
Airflow velocity	8.5	9	9.5	10	10.5	11	11.5	12
Airflow direction	5	7.5	10	12.5	15	17.5		

## Data Availability

Not applicable.
